# Characterization of the complete chloroplast genome of the *Pohlia nutans* M211 from Antarctica

**DOI:** 10.1080/23802359.2020.1721368

**Published:** 2020-02-11

**Authors:** Qing Jin, Liping Zhang, Dan Li, Yingying He, Changfeng Qu, Jinlai Miao

**Affiliations:** a College of Chemistry and Molecular Engineering, Qingdao University of Science and Technology, Qingdao, Shandong, China;; b Key Laboratory of Marine Bioactive Substances, First Institute of Oceanography, Ministry of Natural Resource, Qingdao, Shandong, China;; c Laboratory for Marine Drugs and Bioproducts, Qingdao National Laboratory for Marine Science and Technology, Qingdao, Shandong, China

**Keywords:** *Pohlia nutans* M211, chloroplast genome, phylogenetic tree

## Abstract

The Antarctic *Pohlia nutans* M211 complete chloroplast (cp) genome, sequenced using Illumina NovaSeq PE150, was 125,199 bp in length. It contained 19,836 bp of inverted repeat (IR) regions that separated a large single-copy region (LSC) of 86,738 bp and a small single-copy region (SSC) of 18,580 bp. The whole-genome encodes 132 genes (80 protein-coding genes, 36 tRNA genes, and 8 rRNA genes) and had 29.5% GC content. The M211 was congruent with *Sanionia uncinata* (KM111545.1) according to the Phylogenetic tree analyses.

Antarctica terrestrial ecosystem is cold, limited water, and low nutrient environment, accompanied by sharp temperature changes (Campbell and Claridge 1988; Pearce 2008; Singh et al. 2011). Mosses are the most widespread and abundant photo-synthetically active plant along with and near the islands of the Antarctic continent (Wahrmund et al. 2010). Although moss is a lower group of higher plants in terms of phylogeny (Malenovky et al. 2013), it represents a type of plant that has transitioned from aquatic to terrestrial. The moss is one of the ideal materials to study plant resistance because of the cold, strong radiation of the special living environment, which expected to obtain a number of functional genes with important application value (Moss 2006). Here, we completed the chloroplast genome of *Pohlia nutans* M211 that isolated from the Antarctic land during the 24th Antarctic expedition.

*Pohlia nutans* M211 was preserved in the Key Laboratory of Marine Bioactive Substances, the First Institute of Oceanography, Ministry of Natural Resource, China. The Genomic DNA of M11 was extracted with the SDS method (Lim et al. 2016) and sequenced using Illumina NovaSeq PE150 at the Beijing Novogene Bioinformatics Technology Co., Ltd., moreover, it was assembled with SOAP DeNovo software and predicted with GeneMarkS.

The complete chloroplast (cp) genome of *P. nutans* M211 (GenBank accession MN937553) was 125,199 bp in length and GC ratio was 29.2%. Additionally, it had a large single-copy (LSC) region of 86,738 bp, a small single-copy (SSC) region of 18,580 bp, a pair inverted repeat (IR) regions of 19,836 bp each, and contained 124 genes: 80 protein-coding genes, 8 rRNAs, and 36 tRNAs.

We conducted a phylogenetic analysis to identify the phylogenetic position of *P. nutans* M211, and was based on 14 selected *Bryophytina* chloroplast genome sequences and two species [*Encephalartos lehmannii* (LC049336.1), *Cathaya argyrophylla* (AB547400.1)] as the outgroup to reconstruct by neighbor-joining (NJ) phylogenetic tree in MEGA. The bootstrap value was 1000. The phylogenetic evidence revealed that the *P. nutans* M211 was closely related to *Sanionia uncinata* (KM111545.1) (Figure 1). The result of our study lays the foundation to obtain the functional genes with important application value from mosses.

**Figure 1.**
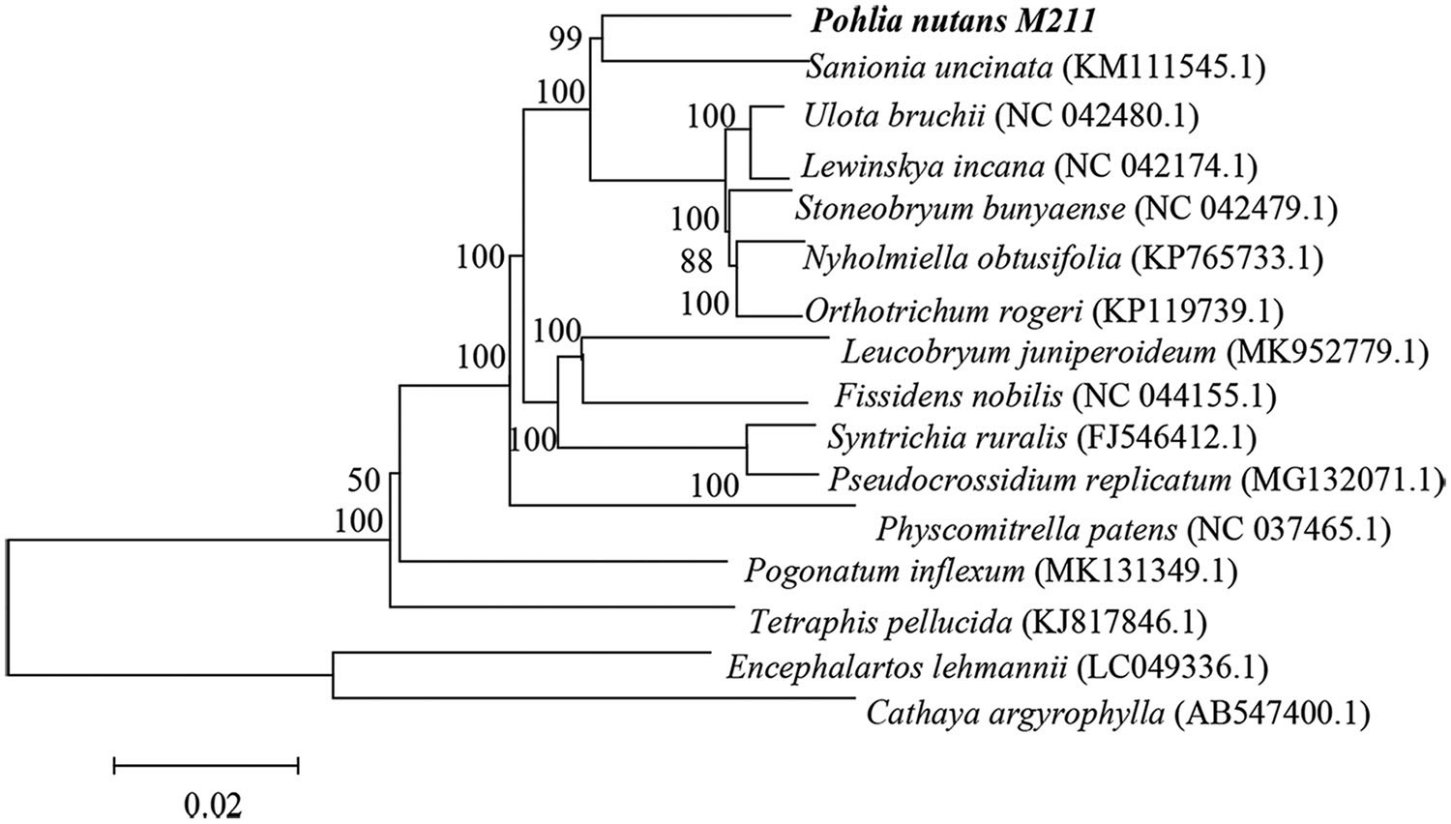
Neighbor-joining (bootstrap repeat was 1000) phylogenetic tree of 16 complete chloroplast genomes.
